# Building Yolŋu Skills, Knowledge, and Priorities into Early Childhood Assessment and Support: Protocol for a Qualitative Study

**DOI:** 10.2196/resprot.8722

**Published:** 2018-03-07

**Authors:** Anne Lowell, Elaine Lawurrpa Maypilama, Lyn Fasoli, Rosemary Gundjarranbuy, Jenine Godwin-Thompson, Abbey Guyula, Megan Yunupiŋu, Emily Armstrong, Jane Garrutju, Rose McEldowney

**Affiliations:** ^1^ Research Centre for Health and Wellbeing School of Health Charles Darwin University Darwin Australia; ^2^ Yalu Marŋgithinyaraw Indigenous Corporation Galiwin'ku Australia; ^3^ Secretariat of National Aboriginal and Islander Child Care Melbourne Australia

**Keywords:** early child development, Aboriginal, culture, internet-based resources

## Abstract

**Background:**

Yolngu or Yolŋu are a group of indigenous Australian people inhabiting north-eastern Arnhem Land in the Northern Territory of Australia. Recent government policy addressing disparities in outcomes between Indigenous and other children in Australia has resulted in the rapid introduction of early childhood interventions in remote Aboriginal communities. This is despite minimal research into their appropriateness or effectiveness for these contexts.

**Objective:**

This research aims to privilege Aboriginal early childhood knowledge, priorities and practices and to strengthen the evidence base for culturally responsive and relevant assessment processes and support that distinguishes “difference” from “deficit” to facilitate optimal child development.

**Methods:**

This collaborative qualitative research employs video ethnography, participant observation and in-depth interviews, involving Aboriginal families and researchers in design, implementation, interpretation and dissemination using a locally developed, culturally responsive research approach. Longitudinal case studies are being conducted with 6 families over 5 years and emerging findings are being explored with a further 50 families and key community informants. Data from all sources are analyzed inductively using a collaborative and iterative process. The study findings, grounded in an in-depth understanding of the cultural context of the study but with relevance to policy and practice more widely, are informing the development of a Web-based educational resource and targeted knowledge exchange activities.

**Results:**

This paper focuses only on the research approach used in this project. The findings will be reported in detail in future publications. In response to community concerns about lack of recognition of Aboriginal early childhood strengths, priorities and knowledge, this collaborative community-driven project strengthens the evidence base for developing culturally responsive and relevant early childhood services and assessment processes to support optimal child development. The study findings are guiding the development of a Web-based educational resource for staff working with Aboriginal communities and families in the field of early child development. This website will also function as a community-developed tool for strengthening and maintaining Aboriginal knowledge and practice related to child development and child rearing. It will be widely accessible to community members through a range of platforms (eg, mobile phones and tablets) and will provide a model for other cultural contexts.

**Conclusions:**

This project will facilitate wider recognition and reflection of cultural knowledge and practice in early childhood programs and policies and will support strengthening and maintenance of cultural knowledge. The culturally responsive and highly collaborative approach to community-based research on which this project is based will also inform future research through sharing knowledge about the research process as well as research findings.

## Introduction

Access to high quality experiences in the early years is widely acknowledged to lead to improved health, education, and social outcomes for young children [1]. However, potential positive outcomes provided by participation in early childhood programs/services are lost when families do not use them [2,3]. The under representation of Aboriginal children in early childhood services in the Northern Territory (NT) of Australia is widely reported [4-6]. Although there is scant research on the reasons for this under representation, particularly for remote Aboriginal families, one reason for resistance may be fear regarding the dominance of Western approaches [7]. Programs that prioritise Western perspectives are criticised for failing to consider local values, goals, domains, languages, learning styles, and learning-teaching paradigms when applied cross-culturally [8-11]. Indeed, recent work in the USA [12], Canada [13,14] and New Zealand [15,16] demonstrates the value of culturally relevant strategies for early child development.

In Australia, there is a growing body of research that points to the need for policies and interventions responsive to distinctive remote Aboriginal cultures and contexts, and the interrelationship of health, wellbeing, and culture [17-19]. Although there has been considerable investigation of Aboriginal parenting practices, few empirical studies of Aboriginal child development have been conducted in Australia. Most of the research related to both child development and parenting has been based on observation by non-Aboriginal researchers [20] or indirect methods such as surveys or questionnaires [1]. Australian research also tends to focus on Aboriginal children in urban areas, and especially on participation rates of Indigenous families in early childhood education [17-19,21]. There continues to be a dearth of research regarding assessment frameworks and outcome measures appropriate to remote Aboriginal populations in Australia [22,23] although there has been recent work to adapt an existing child development assessment tool for remote Aboriginal contexts [23].

The evidence base on which to make decisions about appropriate early childhood interventions (ie, those that are effective for addressing disparities in outcomes between Aboriginal children and other Australian children over the long term) is not strong [24]. While research has demonstrated the promise of some well-known “evidence-based” programs, few have been tested or evaluated over the longer term in an Australian (or Indigenous) context [6,24]. A review of early childhood interventions identified significant gaps in knowledge that create “impediments to implementing interventions more widely and reaping the benefits they promise” [25]. Robinson et al [19] recommend examination of the “cultural logic” and appropriateness of assumptions about child development embedded in such programs and practices.

There are risks in adopting “evidence-based” interventions designed and developed for people in different countries and circumstances, who speak another language and who hold different cultural values. Aboriginal families may reject or not utilise such programs, as noted already in the Northern Territory [26]. Byers et al [27] point out that where world views underpinning an intervention are very different to those of the target group considerable scope exists for misunderstanding, miscommunication of important information and a devaluing of Aboriginal ways of knowing that can reinforce “systematic discrimination and racism” [27]. For example, a recent review of literature on transition to school indicates that where Aboriginal children are assessed using standards and tools developed for mainstream populations their strengths may be overlooked [28].

Early childhood research and education has been critiqued for its reliance on Western methodologies and use of limited epistemological and ontological frameworks [11,29,30]. For example:

Limited experience and knowledge outside the dominant culture has led many well-meaning researchers, policy makers, and local and international government agents to assume that practices within their own highly schooled community define norms for all children’s development, learning and social interaction. This makes it easy to misinterpret the ways of people from many other backgrounds according to a deficit model—to assume that the others have something wrong with them.

11

Findings from the Longitudinal Study of Indigenous Children [31] highlight the importance of using a strengths-based rather than deficit framework. Taylor [32] notes that Aboriginal children’s strengths, such as superior visual-spatial and motor skills as well as ability to assess risk, “rarely appear on ECE checklists or school reports as strengths to be encouraged” [32]. Evans and Myers [33] recommend “interweaving practices that “scientific” evidence would suggest a child needs with effective traditional childrearing practices and beliefs” [33]. Quality early childhood programs that meet the needs of remote Aboriginal children and their families must be informed by local cultural perspectives [24]. Key features of successful early childhood programs and practices consistently recommended by Aboriginal authors and organisations [2,34,35] include that they are responsive, holistic and culturally safe.

The “Growing up children in two worlds” project is being conducted in a remote coastal community in northern Australia with local Indigenous (Yolŋu) community members. The project draws on in depth and situated child development and child-rearing knowledge and practice, providing an evidence base from which early childhood programs, assessment practices and interventions can draw and benefit. It reinforces the value of local cultural knowledges and practice that can foster community ownership and ongoing genuine community engagement, not only supporting local agency but enabling community-controlled decision making [36]. The strengths-based research approach adopts an ethically sound and culturally valid methodology that engages concerned and invested community members in generating and analyzing the knowledge upon which to build a more culturally competent [28] approach to early childhood education and care for Aboriginal children. The project has been funded for a period of three years by Lowitja Institute (2016-18) and extends an initial exploratory project conducted between 2013-15. In summary, this project aims to:

- Privilege Yolŋu (Aboriginal) voices in generating child development and child rearing knowledge
- Identify skills and knowledge (both Aboriginal and Western domains) that Yolŋu families want their children to develop and the strategies they use to foster this development
- Strengthen the evidence base for culturally responsive and relevant assessment processes and support that distinguishes difference from deficit to facilitate optimal child development

The study has received ethical approval from the Charles Darwin University Human Research Ethics Committee.

## Methods

### Study Design

This collaborative qualitative research project draws on culturally responsive methods developed through previous studies of child development and learning, including language socialisation, as well as intercultural communication in Aboriginal health care [37-39]. This approach is closely aligned with elements of constructivist grounded theory [40] in which data collection and analysis occurs simultaneously in an iterative process emphasising theory construction rather than description or application of current theories. The project is a direct response to concerns expressed by community members regarding:

1. Dominance of Western values and practices in early childhood policy and programs
2. Lack of respect and recognition for Yolŋu knowledges, priorities and practices on how best to raise young children and what is important for their development
3. Assessment processes that do not accurately differentiate between “difference” and “deficit”

This project is also a response to the lack of diverse Indigenous perspectives in the early childhood research literature that could inform and improve programs, practices and materials. The research will address these issues and lead to action through providing health and education policy makers and program implementers with new knowledge resources to inform and improve early childhood development assessments and practices.

### Research Team and Governance

The project has been developed in collaboration with senior community members and is a direct response to their concerns. One of the Project Leaders is a senior Yolŋu researcher who has primary control of the research process in collaboration with the two other Project Leaders. As well, emerging Yolŋu researchers participate as members of the research team in data collection, analysis and dissemination activities. All consultation and consent processes as well as research activities are conducted in the preferred language of participants to ensure optimal communication is consistently achieved. Community members and researchers are involved in developing the knowledge-sharing website that is a key component of this project. The strong collaborative approach on which this project is based also engages participants (family members and other key community informants) in interpretation of the data as well as decisions about dissemination. The Aboriginal Project Leader and Partner researchers play a key role in all dissemination activities including authorship of publications and conference presentations.

The project is being conducted in partnership with the Yalu Marŋgithinaraw (an Indigenous community education and research organization). The Balanda (non-Aboriginal) researchers have a long history of collaboration with the community and previous projects have been successfully conducted in partnership with the Yalu to ensure genuine community leadership and engagement is achieved. This collaborative approach and high level of community participation in the project ensures that the research process and specific methods are guided by the Yolŋu researchers and are responsive to community needs and preferences. Collaboration with Secretariat of National Aboriginal and Islander Child Care (SNAICC): National Voice for Our Children (the national nongovernmental peak body representing the interests of Aboriginal and Torres Strait Islander children) is also a critical element of the project to explore broader relevance beyond the study setting and to ensure optimal research translation into policy and practice. SNAICC is providing independent review and advice on the research methodology and findings for the purposes of supporting validation of its robustness and integrity. Importantly, SNAICC review and advice does not seek to impose upon or compromise local Indigenous research methodologies which are integral to the quality of the research process. Rather, SNAICC staff observe the research processes and continuously test research findings with wider audiences for feedback into the project. Through this partnership, SNAICC is in a position at the end of the project to provide strong endorsement of research findings in its role to communicate findings to broader research, community and policy development audiences. The research is supported by two additional groups:

1. *Community Backbone Committee* (Advisory Group) of key community members—this culturally responsive approach developed over many years by the Yolŋu researchers ensures the research process is informed and guided by appropriate Elders and others through continual (often informal) engagement and consultation.
2. *National Backbone Committee* established by SNAICC and comprised of interested groups and individuals who are positioned to facilitate the wider application and dissemination of the project findings and outputs.

### Setting and Participants

The study is being conducted in a large remote community in Northern Australia where Yolŋu (Aboriginal people of Northeast Arnhemland) make up more than 90% of the population [41]. In this region traditional languages as well as cultural knowledge and practice remain strong. English is learned as an additional language at school but is used for limited purposes in the community in interactions with Balanda (non-Yolŋu), for example, in the shop, school and health services. The community is located on an island and accessible only by limited air services or boat and the nearest major town is 500 kilometers away. Participants include children, parents, grandparents and other extended family members involved in six in-depth case studies commenced during an earlier stage of the project (2013-15) that will continue until late 2018. Ages of the six focus children at commencement of the initial project ranged from 1 month to 2 years and all are continuing their participation in the current study. Families from a range of clan groups and key community informants with particular interest and / or expertise in early childhood and identified as appropriate by Yolŋu researchers are being invited to participate in in-depth interviews to further explore the emerging findings from the case studies (approximately 50 participants).

### Data Collection and Analysis

Multiple methods are being used to enable triangulation of data, comparing and contrasting data from a range of sources and perspectives, thus enhancing the trustworthiness and authenticity of findings. These include:

1. Case studies: extensive video recording of six children and their extended families engaging in every day interactions was conducted over two years at 2-3 month intervals as family circumstances allowed. This is continuing at approximately 6 monthly intervals for the duration of this project (providing longitudinal data over 5 years) in response to the participating families’ strong desire to continue this process until their children commence school and beyond. Ongoing interpretation of the video data by family members and Yolŋu researchers, as well as in-depth interviews with participants, identify salient features of child development and child rearing in their specific cultural context as well as relevant strategies to address their needs and priorities. This provides rich empirical data as a basis for the expanded research process and research translation activities that are the focus of this project.
2. Cross-sectional data: the emerging findings from the six case studies are being further explored and expanded through in-depth interviews with interested families from a range of clan groups as well as key community informants identified as appropriate by Yolŋu researchers and the Backbone Committee (Community Advisory Group).

Data from all sources are translated into English and transcribed. An inductive and collaborative approach is used in which categories of analysis are derived from the data to reflect participants’ perspectives and to avoid filtering of the data through a set of restricted and predetermined codes. A qualitative data management program (QSR NVivo 10) is being used to enhance rigor and support the collaborative process of analysis and interpretation implemented with the Yolŋu researchers. Data collection and analysis occurs simultaneously in an iterative process that includes theoretical sampling to elaborate and refine emerging findings [40].

A provisional framework of key features of child development and child rearing, to inform developmental assessment processes and support relevant to the needs and priorities of participants, is under development based on the findings of (1) and (2) and then will be further explored and refined in collaboration with Yolŋu researchers, other key informants, and the Backbone Committees.

### Dissemination and Research Translation

A Web-based multimedia resource integrating the findings is under development by the project team to facilitate transfer of the findings into policy and practice. Elements of the framework based on the findings will be illustrated by salient examples from the video data (selected by participants and used with their informed consent). The website is a mechanism for enabling wide and continued access to Yolŋu perspectives on key aspects of child development and child rearing. This website will provide a training resource to strengthen the cultural competence of staff working in the early childhood field. It will also facilitate cultural maintenance and strengthening of cultural knowledge and practice for Yolŋu across the region with potential wider relevance beyond this specific cultural context. Research dissemination and knowledge exchange will also be achieved with support from the Project Partners (Yalu Marŋgithinaraw and SNAICC), through dissemination of user friendly research reports (written and oral), publications targeting discipline specific journals (ie, health, education, social policy), conference presentations, through the National Backbone Committee (see above) as well as through the project website.

## Results

This article focuses on the research approach used in this project and, therefore, findings will be reported in detail in further publications. However, some initial emerging themes are summarised here. They include: a strong focus from birth on developing children’s Yolŋu identity through understanding of connections to people, place and other elements of the natural world; intensive interaction with, and nurturing by, a wide range of both female and male extended family members; robust stimulation of verbal and nonverbal communication development and recognition of the child as actively engaged in communication from conception. Many aspects of children’s development are closely monitored and regularly purposefully “assessed” by adults in ways that are very specific to the cultural context. Developmental expectations are not age-related and developmental differences are recognised but accepted and valued as individual attributes rather than as deficits. A deeper understanding of diverse cultural strengths and priorities in early child development is crucial to ensure these are recognised, valued and supported. Such evidence may be overlooked or deemed irrelevant through the use of standardised assessment tools but is essential to address the continuing domination of Western values and practices in early childhood policy and practice in remote communities and to ensure “difference” is not confused with “deficit.”

## Discussion

### Principal Findings

The “Growing up children in two worlds” project is a direct response to concerns expressed by community members about the lack of recognition of Yolŋu skills, knowledge and priorities in early child development (see Multimedia Appendix 1). The project provides the opportunity for Yolŋu to influence the ways in which the development of their children is assessed and supported and opportunities for employment are provided to Yolŋu researchers which supports further development of their research expertise. The project will contribute to a deeper understanding of early child development from the perspectives of community members in this cultural context thus enabling more culturally responsive and relevant action to facilitate optimal child development. The collaboration with SNAICC as a project partner will help to share these learnings where they can benefit Aboriginal and Torres Strait Islander children around the country.

The health and wellbeing of Aboriginal people is linked to the degree of control they experience over their lives. Lack of control can lead to high levels of stress which then contributes to other health and social problems [42]. The opportunities offered through participation in this project, to share one’s knowledge and influence policy and practice to be more responsive to one’s needs and preferences, may begin to ameliorate the chronic lack of control experienced by participants and provide a model for others to follow.

Consultations by Guilfoyle et al [43] found Aboriginal families prefer early childhood programs that reflect and incorporate “the culturally based beliefs, values and practices, including child-rearing practices, of individuals, families and communities using that service.” Families, and thus their children, are more likely to use and benefit from such “culturally competent” programs [42]. These can only be developed through strong engagement with the community, such as this project seeks to do.

Aboriginal children are regularly assessed using frameworks that foreground needs and deficiencies over strengths [31]. This “deficit” discourse impacts negatively on their self-esteem and wellbeing. Culturally relevant assessment processes, of the sort this project seeks to facilitate, can more accurately identify their strengths as well as their support needs leading to optimal development and wellbeing.

This project aims to increase understanding of both strengths and challenges related to early childhood in this context, identifying and responding to opportunities to advocate for appropriate action at both policy and practice levels. The findings of this research will provide health and education policy makers and service providers with new knowledge resources to inform and improve early childhood development assessments and support practices. Knowledge exchange activities will be tailored to each target group (eg, teachers, child care workers, health workers, policy makers and governments). As the project progresses the most effective ways to share information will be identified through consultation with each potential user and stakeholder group. Ongoing engagement of Yolŋu researchers and participants in the knowledge production and dissemination processes is a key element of the project.

### Conclusions

Enhanced wellbeing for children is related to their connections to cultural knowledge and practice [44]. Keeping Aboriginal children connected to their culture is seen as a protective factor in their wellbeing and development [45]. This project provides information and a mechanism to enable cultural knowledge and practices to be recognised and reflected more widely in early childhood programs and policies and supports strengthening and maintenance of cultural knowledge. The culturally responsive and highly collaborative approach to community-based research on which this project is based will also inform future research through sharing knowledge about the research process as well as research findings.

## Appendix

Multimedia Appendix 1 Yolŋu perspectives on early childhood.

## Abbreviations

NT: Northern Territory
SNAICC: Secretariat of National Aboriginal and Islander Child Care
